# Family carers and the provision of person-centred dementia care for activities of daily living

**DOI:** 10.1177/14713012241312266

**Published:** 2025-01-06

**Authors:** Chiara Carparelli, Jan R Oyebode, Gerard A Riley

**Affiliations:** 1724 School of Psychology, University of Birmingham, UK; 1905 Faculty of Health Studies, University of Bradford, UK; 1724 School of Psychology, University of Birmingham, UK

**Keywords:** dementia, person-centred care, family carers, activities of daily living, quality of care

## Abstract

Care provided by family members is not always consistent with the principles of person-centred dementia care (PCDC) and interventions to improve the quality of care are needed. A good foundation for the development of such interventions is provided by an understanding of how good and poor care practices are manifested in everyday care, and of the challenges to providing good quality care. Thirty people providing care to a spouse or partner with dementia were interviewed, and asked to describe examples of the care they provided for activities of daily living and the challenges to providing good quality care. Framework analysis was used to guide and interpret the interviews. Interpretation was guided by the VIPS conceptualisation of PCDC which incorporates the principles of *Valuing*, *Individual*, *Perspective*, and *Social*. The quality of care varied, and examples of good and poor care practices are described. The principles of PCDC were sometimes in conflict with one another and with other considerations, such as personal safety and the wellbeing of the carer. Participants were often faced with challenging decisions in which they had to weigh up these different issues. To be credible, guidance for carers need to reflect the complexity of the issues they face.

## Introduction

Person-centred dementia care (PCDC) has been adopted in many countries as an important guiding principle in the provision of care for people living with dementia (Chirico et al., 2021; WHO, 2017). Alongside the ethical value of treating people with the respect required by PCDC, the approach has been shown to have benefits on the quality of life of the person living with dementia (e.g., better emotional wellbeing) and on those providing care (e.g., greater job satisfaction) (Barbosa et al., 2015; Chenoweth et al., 2019). Most research on PCDC has been carried out in the context of dementia care services. However, given its benefits, there have been calls for more research on the provision of PCDC by family carers (Chan et al., 2023; Marulappa et al., 2022; Riley et al., 2020).

*Person-centred dementia care* is a complex concept. In its initial development, Kitwood highlighted the importance of the quality of the personal relationship between the caregiver and care receiver, and that the caregiver must value, respect, and promote the personhood of the care receiver (i.e., their status as an individual, with the same worth and rights as all individuals) (Fazio et al., 2018; Kitwood, 1997). Brooker (2004) provided a way of conceptualising the ideas of PCDC into four general categories, referred to by the acronym of *VIPS*:

• *Valuing*: Carers should value, respect, and promote the personhood of the care recipient, including their autonomy and agency. They should treat the care recipient with respect and in ways that everyone expects to be treated.
• *Individual*: Carers should treat the care recipient as an individual with a unique personal history and personality, with their own wishes, values, and goals.
• *Perspective*: Carers should try to understand situations from the perspective of the care recipient, and act in accordance with that understanding.
• *Social*: Carers should create a positive and supportive social environment in which the care recipient feels valued and appreciated.

Despite such efforts to systematise Kitwood’s original account, the complexity and abstract nature of the construct of PCDC has given rise to much debate about its meaning and adequacy (Dewing, 2008; Nolan et al., 2004). Symptomatic of this uncertainty is the fact that there is considerable variation in suggestions about how to translate the general principles of PCDC into specific guidance about care practice (Hennelly & O’Shea, 2022; Marulappa et al., 2022).

Research on the quality of care provided by family members, and on how well it conforms with the principles of PCDC, is relatively limited. Some of the research has considered a broad range of carer actions in order to categorise them from a PCDC perspective (e.g., Leocadie et al., 2020; Perry & O'Connor, 2002). Other studies have focused on a narrower range of carer actions, such as whether families involve the person living with dementia in decision-making (e.g., Larsson & Osterholm, 2014; Miller et al., 2016; Samsi & Manthorpe, 2013), or how they respond to challenging emotions or behaviours (Leung et al., 2021; Riley et al., 2020). Although the care provided by families in these qualitative or mixed-methods studies is often of high quality, actions that are inconsistent with the principles of PCDC have also been described (e.g., Chan et al., 2023; Larsson & Osterholm, 2014; Miller et al., 2016; Tyrrell et al., 2006). Quantitative studies suggest that such actions are not infrequent. For example, in a study of carers in South Korea, Kim et al. (2018) found that over half the sample admitted psychological abuse over the previous 3 months (e.g., shouting, threats) and one in seven admitted physical abuse. This evidence suggests that family carers may sometimes need intervention and support to enable them to deliver a good standard of care.

A major category of carer activity that has received relatively little attention in relation to PCDC is support for activities of daily living (i.e., activities completed on a regular basis to meet one’s basic needs, such as dressing and food preparation, and more complex ones required for community living such as shopping and managing money). Although it has not been their primary focus, some of the studies referenced in the previous paragraph include material relevant to this topic. For example, some describe how family carers simplify activities of daily living to ensure that the person living with dementia retains agency (e.g., offering a choice between two alternatives rather an open-ended choice) (Chung et al., 2017; Perry & O'Connor, 2002; Phinney, 2006; Samsi & Manthorpe, 2013). Vikstrom et al. (2005) focused specifically on the quality of family care for activities of daily living, by observing couples making a simple meal together. They did not explicitly use the conceptual framework of PCDC to evaluate their findings but many of the observed activities can be classified in terms of their consistency with the principles of PCDC. Some of the activities were consistent, such as simplifying tasks so the person living with dementia could be actively involved in its completion (*valuing* principle) and taking various steps to create a supportive social environment (*social*). Other actions were inconsistent, such as taking over the task (*valuing*), or giving complex and unclear instructions (*perspective*).

In summary, PCDC has ethical and practical benefits but family carers sometimes provide care that is inconsistent with PCDC. Interventions to support family carers to provide better quality care would therefore be useful. Because of the complexities surrounding the concept, how the principles of PCDC should be translated into practice is not always clear. This uncertainty is magnified by the lack of research on the quality of care provided by family carers. Assistance with activities of daily living is a major part of the care provided by family carers but is under-researched.

In developing practical guidance and support for family carers, an important starting point is an understanding of how the principles of PCDC should be translated into everyday caring. To address the uncertainty about this translation, one approach is to obtain from family carers an account of specific actions they take in everyday caring, and to classify these according to whether they are consistent or inconsistent with PCDC. This approach has an advantage over generating guidance from a theoretical starting point. The provision of family care often takes place in a complex and demanding situation. Good care practices that have been tried and tested in such an environment are more likely to be adopted by family carers than suggestions generated from a theoretical or professional perspective (Phinney, 2006; Vikstrom et al., 2005). Furthermore, a focus on the accounts of family carers also provides an opportunity to explore the challenges they experience, in a complex and demanding situation, of trying to deliver good quality care. Interventions that address these challenges are more likely to be effective. Finally, an account of the specific ways in which less person-centred practices are manifested in everyday family care is also important because these practices, too, need to be identified and addressed within the intervention.

### Aims of the present study

The purpose of the present study, therefore, was to obtain from family carers an account of specific activities they perform in assisting with activities of daily living, and to classify these according to whether they are consistent or inconsistent with PCDC. The VIPS framework was used to evaluate the activities in these terms. The study also aimed to explore the challenges that the participants faced in providing PCDC-consistent care.

## Method

Qualitative methodology was used to address the aims of the study. Participants were interviewed, and transcripts of the interview were analysed using framework analysis. The transcripts were also analysed quantitatively to investigate the relationship between PCDC and the quality of the relationship between the care giver and care receiver. The methodology and findings of the quantitative component of the study are reported elsewhere (Carparelli et al., 2024b).

Ethical approval for the study was provided by the Science, Technology, Engineering, and Mathematics research ethics committee at the University of Birmingham, UK, reference ERN_18-1795C. Participants provided verbal consent that was fully informed and audio-recorded.

### Participants

Participants were recruited through *Join Dementia Research*, an online service provided by the UK National Institute for Health and Care Research that allows those living with dementia and their families to register interest in participating in dementia research.

To be eligible to participate, the carer needed to meet the following criteria:

• Spouse/partner of someone diagnosed with dementia at least 3 months previously
• Currently providing help to partner for completion of activities of daily living
• Lived with their partner for at least 5 years before the onset of dementia
• Did not provide substantial care to their partner prior to the onset of dementia
• No co-existing learning disability, major mental health disorder or other condition that may impact on their ability to take part

The final sample consisted of 30 people, 10 men and 20 women who were carers for their heterosexual partner. Ages ranged from 56 to 88 (mean = 73). With one exception, all identified as White British. The length of time they had lived together ranged from 11 to 64 years (mean = 38). The length of time since diagnosis ranged from 1 to 9 years (mean = 3), although most had been providing care for some time before an official diagnosis was given. Alzheimer’s Disease was the most common diagnosis (19 cases), followed by vascular dementia (5) and mixed dementia (5), with one case being ‘dementia not otherwise specified’. Information about the diagnosis was obtained from the participants themselves. Most participants lived at home with their partner but, in three cases, the partner now lived in a nursing home. These participants still assisted with activities of daily living during their visits. Comparison of their accounts with those of the other participants did not reveal major differences, and so they were retained in the sample.

### Procedure

Due to the risks associated with the COVID-19 pandemic, participants were interviewed via Zoom or telephone, depending on their preference. Interviews took 30–90 minutes, and were audio-recorded and transcribed. After completion of the interview, participants completed the Birmingham Relationship Continuity Measure (Riley et al., 2013) for the quantitative component of the study.

A detailed schedule was used to guide the conduct of the interviews. Prior to interview, participants were asked to identify some activities of daily living in which their partner was still engaged but for which they needed supervision or support from the participant. In the interview, the participant was asked to describe in detail how these tasks were carried out, what support or supervision they provided during these tasks, why they provided support in that way, and what challenges there were to providing the support in that way. The interview also explored other issues relating to the participants’ understanding of the concept of PCDC, the rationale underlying the decisions they made about providing care, and advice and guidance they had received about providing care. Findings related to these issues are described in the Supplemental online material.

### Data analysis

Framework analysis was employed (Ritchie & Spencer, 1994). The main advantages of this for the present study are that it allows codes to be based on prior research and conceptualizations, as well as being generating inductively; it is appropriate for qualitative research seeking answers to specific research questions (as opposed to more open-ended exploration); and it is suitable for the analysis of larger datasets (Goldsmith, 2021; Srivastava & Thomson, 2009).

Framework analysis involves the stages of familiarisation, establishing the thematic framework, indexing, charting, and mapping and interpretation (Ritchie & Spencer, 1994; Srivastava & Thomson, 2009). The first and third author initially familiarised themselves with the first three transcripts by reading them through and recording preliminary ideas. An initial thematic framework was established based on these preliminary ideas and on the VIPS categories. Using this initial framework, the first three transcripts were then indexed by the first and third author – that is, interview extracts corresponding to the themes were highlighted on the transcripts. Points of disagreement were discussed and resolved, and new codes, based on the interviews, were added to the framework. These new codes were all subthemes of the VIPS themes already present in the initial framework. The first and third author then independently indexed the remaining transcripts, using the revised framework. In the next stage (charting), indexed excerpts from all the transcripts were arranged under the themes. Finally, the charted data were mapped and interpreted through discussions involving all the research team, with the aim of creating an overall account of the data that addressed the research aims.

### Trustworthiness

Several steps were taken to enhance trustworthiness, addressing the issues of credibility, transferability, dependability, and confirmability (Lincoln & Guba, 1985; Shenton, 2004). An established qualitative methodology was used, and its application documented in this paper. This included using a detailed interview schedule. A relatively large number of participants took part, allowing for cross-confirmation and the exploration of differences. In writing up the study, care was taken to provide sufficient excerpts from the interviews to support the account. Analysis was undertaken independently by both the first and third authors prior to agreeing on the final interpretation. As part of the quantitative component of the wider study, the first and third author independently rated excerpts in terms of whether the action was more or less person-centred and which VIPS categories were applicable, and these ratings showed good agreement (Carparelli et al., 2024b). Excerpts where there was disagreement between the raters or other uncertainty about their interpretation, have not been used to support the account. Table 1 provides information about earlier qualitative research in which similar themes to those reported in the present study were described. The second and third authors have prior experience of working as clinicians in dementia services, and of research on the issue of person-centred care and family relationships. The study was completed as part of a doctorate obtained by the first author who had experience of working as an advisor for a dementia charity. Meetings of the research team were used to reflect on developing ideas and potential biases. To assist transferability, the sampling method has been described in detail, and demographic and dementia-related information has been provided about the participants.

**Table 1.** Summary of findings on actions consistent or inconsistent with PCDC principles.

*Valuing - consistent*
• Minimising assistance
- Partial reminders and routines rather than telling someone to do something
- Verbal guidance rather than physical assistance
- Questions and prompts rather than direct instructions about steps in the task
- Letting partner work out solution to errors or difficulties themselves, rather than doing it for them
- ‘Hands-off’ approach – only providing help when asked; letting person complete task unsupervised and only checking later
*Similar findings:* Chung et al. (2017); Guðnadóttir et al. (2023); Merrick et al. (2016); Vikstrom et al. (2006)
• Giving choices
- Even if partner may not respond
- Even if the partner makes a inappropriate choice
*Similar findings:* Larsson & Osterholm (2014); Samsi and Manthorpe (2013); Sinclair et al. (2018)
• Keeping partner informed and involved
- Using whiteboards or diaries to go through the plans for the day
- Informing them about activities of daily living even then they no longer have any substantial involvement
*Similar findings:* Jansson et al. (2001); Vikstrom et al. (2005)
***Valuing – inconsistent***
• Taking over activities of daily living even when partner still capable
• Not giving choices
• Not keeping partner informed
• Limiting independence because of concerns about consequences of errors
- Occasionally in relation to relatively minor consequences
- More often in relation to potential for serious harm or loss
- Sometimes restrictions were only partial, allowing partner some independence within boundaries designed to limit the loss
- Use of deceit to prevent engagement in risky activities
*Similar findings:* Berry et al. (2015); Chan et al. (2023); Perry and O’Connor (2002); Vikstrom et al. (2005, 2008)
***Individual – consistent***
• Prioritising involvement in activities known to be important and meaningful to the partner, based on their past preferences
• Providing help based on knowledge of the partner’s longstanding emotional responses to situations
*Similar findings:* Chung et al. (2017); Jansson et al. (2001); Perry and O’Connor (2002)
***Individual – inconsistent***
• Absence in some interviews of actions consistent with the principle
***Perspective – consistent***
• Understanding the cognitive challenges of the task and making adjustments
- Simplifying the tasks by using specialised equipment or other adjustments
- Written or pictorial instructions
- Simplifying instructions
- Simplifying choices
- Written reminders and instructions
- Reducing environmental distractions
- Keeping everything in a set place and maintiang routines
*Similar findings:* Chung et al. (2017); Merrick et al. (2016); Perry and O’Connor (2002); Phinney (2006); Samsi and Manthorpe (2013); Small et al. (2003); Vikstrom et al. (2005)
• Understanding partner’s emotional response to situation and responding empathically
- Varying degree of assistance depending on current emotional state of partner
- Wording reminders and instructions sensitively
- Taking over a particular activity of daily living to avoid upset
- Adopting a ‘hands-off’ approach to providing help
- Avoiding criticism of performance or choices
*Similar findings:* Perry and O’Connor (2002); Vikstrom et al. (2005)
***Perspective – inconsistent***
• Absence in some accounts of actions consistent with the principle
***Social – consistent***
• Avoiding negative feelings during task completion
- More assistance when partner struggling with a task
- Avoiding insensitively worded guidance
- Avoiding confronting partner with error – either accepting the task is not completed to usual standards, or correcting errors later in absence of partner
- Words of comfort if partner is struggling
- Using humour to counteract potential distress
*Similar findings:* Chan et al. (2023); Chung et al. (2017); Guðnadóttir et al. (2023); Hickman et al. (2020); Perry and O’Connor (2002); Vikstrom et al. (2005, 2008)
• Creating a positive atmosphere
- Using humour
- Positive feedback about performance
- Doing activities of daily living together
*Similar findings:* Hellström et al. (2007); Phinney (2006); Vikstrom et al. (2005, 2008)
**Social - inconsistent**
• Harshly-worded commands or corrections
• Aruguing and losing temper with partner
*Similar findings:* Chan et al. (2023); Kim et al. (2018); Leocadie et al. (2020); Vikstrom et al. (2005)

## Findings

As noted earlier, the partners of three of the participants had moved into residential care. These participants still assisted with activities of daily living during their visits to their partner and comparison of their accounts with those of the other participants did not reveal major differences. They were accordingly retained in the sample.

The VIPS principles were used to organise the description of actions. In all cases there were examples of actions consistent with the principles. In the case of the *valuing* and *social* principles, there were also clear examples of actions that were inconsistent with the principle. Examples of actions inconsistent with the *individual* and *perspective* were less frequent, but inconsistency with these principles could be inferred from the absence in the interview of any actions consistent with the principles. The findings are summarised in Table 1, which also provides references to earlier research that has reported similar outcomes. A final section reports on the challenges experienced by participants in providing care that was consistent with the principles.

### Valuing – consistent actions

A key component of the *valuing* domain relates to promoting the agency and autonomy of the person. Translation of this general principle into activities of daily living practice was evident in efforts to minimise the degree of assistance given to partners, to give them choices, and to keep them informed.

#### Minimising assistance

Activities of daily living assistance took the form of prompts to undertake a task, help with completing steps in the task, and help to put things right if errors were made. Many partners used forms of assistance that required a proactive rather than passive response on the part of the partner. In terms of prompts, there was a preference for partial cues over direct instruction to do something. For example, rather than simply telling the partner to take their medication, some participants would place it in a prominent place to serve as a reminder to take them. Routines also served as scaffolding to support independence:

I don’t need to remind him, he knows routine. He knows that at breakfast he takes one lot [of medication], and then he knows when he's going to bed, he takes another. (13 – numbers are used to identify individual participants)

In terms of assistance in completing steps of the task, some expressed a preference for verbal rather than physical assistance because the partner exercised more autonomy in responding to verbal assistance:

I do help now with [getting dressed] but initially, for quite some months, I would guide him rather than actually physically help him dress. We managed, and I was keen for him to keep on going. (22)

When verbal guidance was given, questions and prompts to think about the situation were preferred over direct instruction because of the increased control this gave the partner:

How do I phrase it? ‘Do you think it would be a good idea for you to take that one off and put a clean shirt on. Shall we do it?’…I ask him, rather than saying you should… because I think, once again, it’s putting him in control of his own choices and life. (25)

If errors were made or difficulties encountered, some participants preferred to point it out and let the partner work out how to correct it, with guidance if necessary, rather than sorting it out themselves.

Some participants described a ‘hands-off’ approach to assistance. For example, some would try to provide help only when asked:

Try to step back if I can. It is difficult, but I try to step back and say, ‘right you try, and let me know when you need any help’. (20)

Another strategy to promote the autonomy of the partner was to leave them to get on with the activity alone and to check later whether things had been done correctly:

He does all the things like online banking…but I have to look at my iPad every day [to check] that he hasn’t done anything daft. (9)

#### Giving choices

Another category of maintaining independence and control was to involve the partner in making choices. Participants encouraged their partner to contribute to decisions such as what to have for a meal, what clothes to wear, and what to buy. Some considered it important to offer choice even when they were unlikely to get a clear response or when they disagreed with the choices being made:

If he was lying in bed all day long completely out of it - which he will get like that - then it’s different, it’s different how you speak with him. But I try and give him the opportunity [to make a choice] on the off-chance that he understands… I still ask him. (11)

I shouldn’t impose things on him even if he’s not making the best choice. I still don’t feel I should impose it. (2)

#### Keeping partner informed

Behaviour in accordance with the *valuing* principle was also evident in the steps that participants took to keep their partner informed about what was happening. Some would use diaries or whiteboards as the basis for talking through the plan for the day. Providing his partner with this context was viewed by one participant as a helpful way of motivating her to do activities of daily living:

I’ve learned it’s good for [her] to know what is going to happen. So [I] start by saying, ‘Well we’re going to have lunch with some friends’ or whatever you know, ‘but to get there, we perhaps need to go up and have a shower, and let’s get ready’. (21)

Even when the partner was no longer involved to any great extent in an activity of daily living, some considered it important to keep them informed about what was happening:

Because, as I actually do all the shopping and things like that, I write everything down what I get and I show her the list before I go. (23)

As with giving choices, one participant expressed the importance of doing this even if there was some doubt about her partner’s ability to retain the information:

I’ve been trying to sort the finances for it and do involve, well, keep him up to speed with that. I don’t know whether he can retain it, but I feel I need to keep him informed. (18)

### Valuing – inconsistent actions

#### Taking over activities

For a few participants, promoting independence was not a major priority and they had taken over most activities of daily living activities even though it seemed their partner was still capable of some degree of involvement:

I think it came to a point where I thought it was going to be quicker and easier if I do it, than if I have to stand over him while he’s doing it. (30, referring to food preparation)

#### Making choices on their behalf and not keeping them informed

Some participants made most of the decisions for their partner even when the partner appeared capable of expressing a choice, and providing choice was not prioritised or appeared to be a token gesture:

I’ll say, ‘Would you like to do that?’. Normally, the answer’s no. But we might do it anyway. If I’ve decided we’re going to do it, we probably will do it. (3)

Keeping their partner informed was also not a concern for some:

It’s no good explaining to her what they are, because she don’t accept she’s got these conditions. (4, when asked if he explained why he was giving his wife tablets)

#### Limiting independence

Independence was sometimes curtailed to avoid negative consequences. These consequences could be relatively minor:

If we’re going somewhere then I have to [choose his clothes] and make sure he’s dressed properly, [otherwise] he would just wear the same old things all the time. (12)

More usually, though, the concern was about serious loss or harm. Participants restricted freedom in relation to activities like driving, medication, using equipment, and spending money:

I just get everything ready, get it out and give it [medication] to him because that’s something I can’t afford to have go wrong, you know, I don’t want him to be overdosing or missing doses. (18)

Sometimes the restrictions were only partial and intended to limit potential for loss while still allowing the partner some freedom. For example, one common strategy was to allow the partner to continue using bank card payments but to reduce the risk of loss by capping the amount of money contained in the account:

I’ve taken a lot of [money out of his bank account]. So if anything goes wrong, there’s not as much in the account if he was to lose a card or something like that. (10)

Sometimes, however, the restrictions were more limiting and involved the use of deceit. Examples included hiding bank cards, locking the computer to prevent online shopping, hiding car keys to prevent driving, and parking the car away from the house so that the sight of it would not act as a prompt to go for a drive. These actions clash with the *valuing* principle in terms of both limiting autonomy and using deceit, given that the principle is also about treating the care recipient with respect and in ways everyone expects to be treated. Another concern about consequences that led participants to restrict autonomy was the impact of mistakes and difficulties on the psychological wellbeing of the person living with dementia. This is discussed later in relation to the *social* principle.

### Individual– consistent actions

The *individual* principle is about caregivers treating the care recipient as an individual with a unique personal history and personality, and their own wishes, values, and goals. In the present context, this was evident in the participants using their knowledge of their partner and their life together to inform the decisions that they made about providing help.

#### Partner’s longstanding interests

Some participants put extra effort into maintaining their partner’s involvement in activities and roles that were perceived as important to the partner, even when the partner had begun to struggle with the activity. For example, one participant involved her partner in gardening, even though physically he was not able to contribute much, by asking him questions about what she should do in the garden “because he’s always done it in the past and he’s a naturalist” (25). Another encouraged her husband to continue using a bank card, despite her concerns about him losing it, because “he likes to think if we go out, he could perhaps pay for, you know, the meal or something like that” (11). Conversely, participants put less effort into maintaining involvement in tasks that they knew their partner had never had any major role in and in which they showed little current interest. For example, one participant (30) did not encourage her husband to get involved in cooking because, before the dementia he had only occasionally been involved, and he currently showed no interest in maintaining what little involvement he previously had.

#### Partner’s longstanding emotional responses

Participants also used knowledge of their partner’s longstanding emotional responses to situations to understand their partner’s emotional response to the current situation, adjusting the support provided in accordance with this understanding. Examples of this will be provided in the next section about *Perspective*.

### Individual – inconsistent actions

There were no clear specific examples of actions inconsistent with the *individual* principle. However, some interviews lacked any examples that were consistent with the principle, suggesting that some participants took less account of their partner’s individuality when providing care.

### Perspective – consistent actions

The *perspective* principle is about carers trying to understand situations from the perspective of the care recipient, and acting in accordance with that understanding. Participants spoke of making adjustments in response to their understanding of both the cognitive and emotional challenges of the situation.

#### Responding to cognitive difficulties

Adjustments included simplifying the task, providing written or pictorial instructions, simplifying the instructions/choices, and removing environmental distractions, and maintaining routines. Simplifying the task involved using adapted equipment and materials, or devising other strategies, to reduce demands on the partner’s cognitive abilities. Examples of adaptations included a push-button shower to enable the partner to shower independently, a dosette box to enable the partner to stay in control of taking their own medication, ready-made cake mixes to enable the partner to continue baking, a voice-operated TV, and software to simplify the use of passwords. Adaptations were also used to enable the partner to continue with potentially risky activities. Tracking devices were used by several participants to give their partner the freedom to go out alone:

I do let him take the dog for a walk through an area, which is very secure and everybody knows him so and I’ve also got some trackers. I’ve got a tracker for him and I’ve got a tracker on the dog. (22)

Adaptations were also used to enable the partner to maintain some control over the use of money. These included making sure that the partner had the right amount of cash when going to a shop and simplifying the PIN number to make it easier to remember.

Written instructions were often used to circumvent memory difficulties, both to remind the partner to do something (e.g., via whiteboards or post-it notes, reminders on their mobile phone) and how to do it (e.g., step-by-step recipe cards or instructions for using the TV remote control). Participants also described using simplified instructions or simplified choices because their partner could not cope with too much information at once:

Quite often it’s saying things at [each] step rather than saying ‘you do this then you do that then you do this’. (30)

To address attentional difficulties, some participants described reducing environmental distractions:

Another thing is that he finds he can’t deal with…a lot of noise, or a lot of things moving quickly. So sometimes he’ll say that, if I’m in the kitchen with him, that I’m moving about too quickly. That’s something we have to try and adjust to. (24 with reference to cooking)

Other strategies to reduce the cognitive challenges of activities included keeping things in a set place, and maintaining routine. One participant noted that the routine not only prompted her partner to do something, but also helped him to remember how to do it:

It’s only because that’s his routine, you know, routine that’s been set for 50 [years] and he knows how to do that, but he can’t seem to relate to doing it at different times of the day. (13, discussing the fact that her partner made coffee without help first thing in the morning, but struggled if she asked him for a coffee at other times of the day)

Another action consistent with the *perspective* principle was varying the amount of assistance according to the partner’s cognitive status. Some were able to empathise with whether the partner was perceived to be having a ‘good’ or a ‘bad’ day, and adjusted the degree of assistance accordingly:

I mean, sometimes he can just choose. It depends; it can be very day to day. Sometimes you wouldn’t even know that he has struggles and he’ll just do it and that’s it; but other days, a confusion comes down and he really needs help. (20)

#### Responding to emotional impact on partner

A person-centred *perspective* was also evident in understanding the emotional impact of the situation on the partner and responding empathically. Some participants varied the amount of assistance they provided for activities of daily living in accordance with their perception of their partner’s emotional state at the time:

Her ability to do things for herself is directly impacted by how much stress she’s under, and some of that is things which are stressful and some of that is things that she perceives to be stressful and she gets herself into a state. So, it just does depend what mood she’s in. So, it can range from I don’t have to do anything, to I have to help her get dressed. (8)

Some participants also showed appreciation of the general emotional impact on their partner of being dependent. They were aware of how reminders, instructions, and corrections might come across to their partner, and the importance of wording them respectfully and sensitively. Direct reminders or instructions were avoided, and indirect methods were favoured, such as asking questions or making suggestions:

If she’s trying to do something and I take it over – that must be endlessly annoying when somebody says, ‘Oh it’s like this, you got to do it this way’. You’re muddling through, yeah, and then you’ve then got somebody coming up behind you saying, ‘Are you doing that right? You got to do it this way’…I try hard not to do that. (21)

This aspect of perspective-taking was often informed by the participant’s shared history with their partner and understanding of their preferences and values (relating to the *individual* principle):

I know the kind of things that he will find patronizing or difficult, so I try not to do that, and I try to encourage other people not to do that. (6)

Awareness of the impact of dependency sometimes led the participant to relieve their partner of responsibility for a specific task. For example, one participant gave up trying to encourage her partner to take his medication because of her concern that he would feel harassed:

If I said, ‘Go and take your medication’, and he’ll say, ‘yes, I will’. But then, unless I keep saying ‘Go and take it now’ [he won’t], and that can, you know, that feels like nagging sometimes…So, I just go and get it and say, ‘Here’s your medication’. (6)

Conversely, assistance was sometimes held back to avoid upsetting the person:

If sometimes if I try to help too soon, he could get a bit frustrated and short tempered, it upsets him more. So, then, you know I usually try and wait. (24)

Some participants also avoided direct criticism of what the partner had done or the choices they made, and tried to guide them indirectly:

She’ll have four or five different choices, and I might just suggest ‘well you look really good in all of those but this one I like, that goes nicely with what you’ve got on’. Do it with compliments rather than, you know, ‘that’s a stupid choice, you look ridiculous’. (21)

### Perspective – inconsistent actions

Specific instances of not following the principle were infrequent. However, the absence in some interviews of any specific instances of actions consistent with the principle suggested variation across participants in this respect. For example, not all participants discussed how their partner’s cognitive difficulties necessitated adaptations and strategies to promote their independence, and not all articulated an understanding of what the emotional impact of the loss of independence might be.

### Social – consistent actions

The *social* principle is about carers creating a positive and supportive social environment in which the care recipient feels valued and appreciated. This was evident in participants doing things to promote positive feelings and avoid negative feelings in their partner during activities of daily living. Examples of divergence from the principle was evident in interpersonal behaviour that was likely to have a negative impact on the partner’s emotional state.

As highlighted in the section on *perspective*, negative feelings were avoided by providing more assistance when a participant perceived that their partner was struggling and getting upset, and by avoiding insensitively worded guidance. Rather than correcting error and causing upset, participants sometimes let their partner carry on doing something in the wrong way, and accepted that the task was not completed to usual standards:

He’ll make a start of doing it, then he loses completely the reason why he’s doing it, and he’ll just stand and stare at it, which usually means that I get very cold cup of tea or a cup of tea with a spoonful of coffee in it. (22)

Alternatively, the participant would correct mistakes later when their partner was not there, to avoid the person being confronted with their failure:

You know, if I think something’s dirty I will either wash it later, or just ignore it, or whatever. (14, discussing her husband’s dishwashing)

When errors did occur, some participants would make a point of offering words of comfort and support, suggesting a less upsetting appraisal of the reasons for the difficulties:

And I try and provide emotional support and comfort to him; and explain that it’s not him, it’s the fact that he has Alzheimer’s disease. (6)

Participants also sometimes used humour to try to mitigate any negative reactions to mistakes:

I say…‘That’s different - wasn’t expecting that to be there’...We have a bit of a laugh about it. (21 on finding cutlery and other things in odd places)

As well as protecting the person from negative feelings, some participants tried to create a more positive atmosphere for the completion of activities of daily living. Again, humour played an important role in this:

We’ve always had humour and we’re kind of in this together, and I think you’ve got to keep that kind of attitude. (18)

Participants also described giving their partner positive feedback such as words of encouragement, thanking the partner for doing the task, and giving compliments about a job that was well done. Doing activities of daily living together was seen by some as a way of creating a positive and supportive context for the partner:

To make sure we get a good outcome, we’ll probably do things together - whether it’s hanging up the washing, going to get something from upstairs, we’ll perhaps both go up to make sure he’s remembered what it is he’s gone up to get…It’s a confidence thing for him. (19)

### Social - inconsistent actions

Some participants described behaviours that appeared to run the risk of causing negative emotions in their partners. These included what appeared to be harsh commands or corrections to their partners:

I ask him to get the vacuum cleaner occasionally…but he has no clue what he’s doing, not plugged in, or it’s not switched on, and there he is trying to vacuum. And I’m saying to him, ‘Can you hear anything? No? Well the vacuum cleaner makes a noise doesn’t it? It sucks and you haven’t even got it plugged in. So how’s it going to work?’ And he gets flustered and ‘oh, oh, oh’. (13)

Activities of daily living could often trigger an argument between participants and their partners, and many described losing their temper with their partner when they refused to do something, or struggled with the task. Most expressed regret for behaving in this way:

He wouldn’t get up and out of the chair and I’m afraid I did lose my temper with him… And I think I could have done things differently there…And I’m afraid I did, I grabbed a hold of his arm and said ‘get up’. (27)

### Challenges of providing PCDC-consistent care

As well as enquiring about strategies for assisting with activities of daily living, the interviews also explored some of the challenges participants experienced in trying to provide care for activities of daily living that was consistent with the VIPS principles. A major challenge was that the principles sometimes clashed. For example, there was often a clash between increasing the assistance provided in order to avoid distress and offering the minimum assistance necessary in order to maximise independence. In this situation, the participants were faced with choosing between the promotion of agency and independence (*valuing*) versus protecting the partner from distress (*social*) and understanding the situation from their partner’s perspective (*perspective*). Participants described the dilemma of trying to get the balance right:

It’s always that balance between letting him try and work it out himself in the hope that it, you know, maintains that bit of independence, and not just letting him struggle and get upset. (6)

Another example of a clash between the principles was the dilemma described by one participant of whether to let her partner choose his own clothes and dress inappropriately (*valuing*) when she knew how much importance he placed on his appearance (*individual*).

You don’t want him to still be in his pyjamas. So, it is something that maybe I don’t need to do, but I do have this feeling that… for his pride, because he always looks so nice and took pride in it. I mean he’s a nice-looking man, and so it’s a bit of a dilemma really how much to interfere, and how much just to let him and get on with it. (14 talking about choosing what clothes her husband wore)

Another major challenge was that the VIPS principles sometimes clashed with other important considerations. As described earlier, concerns about serious harm and loss often led participants to curtail freedom and choice (inconsistent with *valuing*) or use deception (also inconsistent with *valuing*) (e.g., hiding car keys and bank cards). Participants’ own needs and wellbeing also needed to be considered: Encouraging independence can be time-consuming and effortful. When participants felt stressed or tired, or when there were other demands on their time, they sometimes took over tasks to save on time and effort (inconsistent with *valuing*):

I think the only time [that I don’t encourage him to do it himself] is when there’s perhaps several things have happened, perhaps I’m tired myself. That’s a tremendous thing really, if you’re tired yourself, you really can’t be bothered and you’re tired and you try to sort something out and then something else happens and you’ve got to try and sort that out first. (30)

One participant also highlighted the importance of not overburdening her partner and expecting him to do too much for himself, given how challenging he found the tasks.

I try and make him do as much as he can for himself without forcing him to do too much. (27)

Another challenge noted by participants was how easy it is to forget to provide good care, particularly when the alternative is to complete the task more quickly and more easily oneself:

It’s so much easier to do things yourself than ask the person to do it, but you have to sometimes stop yourself. It’s easier and quicker if I do it, but then that’s not always the point. (20)

## Discussion

The main aim of this study was to describe, within the context of support for activities of daily living, some specific examples of actions that were consistent or inconsistent with the principles of PCDC. Earlier studies have similarly reported on activities of daily living -focused actions consistent and inconsistent with PCDC (see Table 1), but this study has provided a more comprehensive systematic account through using the VIPS framework.

Another aim was to explore the challenges faced by participants in providing care consistent with the VIPS principles. The principles sometimes clashed with one another and with other considerations such as the avoidance of harm and loss, and the participant’s own wellbeing. These clashes gave rise to dilemmas about the appropriate course of action. The challenge was heightened by the fact that sometimes carers had to make rapid decisions in changing circumstances, such as when they were making moment-to-moment judgements about the emotional state of their partner and whether they needed to step in and provide more assistance. The fact that there is no correct solution to some of these dilemmas increases the challenge. For example, it is not clear whether one should prioritise encouraging independence or avoid one’s partner getting distressed. Previous research has similarly highlighted the complexity of decisions faced daily by family carers (Jansson et al., 2001; Leocadie et al., 2020; Samsi & Manthorpe, 2013; Vikstrom et al., 2008).

The present findings indicate the need for family carers to receive more support in providing good quality care in relation to activities of daily living. Although there were many examples given of help that aligned with the principles of PCDC, there were a substantial number that did not. Previous studies have similarly highlighted that the care provided by family members is not always of a satisfactory standard (e.g., Chan et al., 2023; Hirschman et al., 2005; Kim et al., 2018; Larsson & Osterholm, 2014; Miller et al., 2016). Furthermore, in additional findings of the study reported in the supplementary online material, participants highlighted the lack of guidance they had received about providing help with activities of daily living; they showed limited explicit awareness and understanding of the principles of PCDC; and only two said that they intentionally tried to implement those principles in practice. Family carers themselves have also expressed a wish for more guidance about assisting with activities of daily living (e.g., Nichols et al., 2009).

Despite the very large number of previous studies that have evaluated interventions for family carers in dementia (a recent meta-review identified over 500 – Cheng & Zhang, 2020), relatively few have aimed to improve the person-centredness of care provided by the family (Marulappa et al., 2022). When person-centred care is addressed, it is typically in the context of multi-modular interventions that deal with many aspects of care. Not all of these include specific modules about activities of daily living (e.g., Boots et al., 2018), and a search identified only two that did. Gaugler et al. (2015) evaluated an online training programme (CARES™) for family carers adapted from a programme devised for paid carers. The programme includes a module about assisting with activities of daily living, and its aims include increasing the understanding and use of the principles of PCDC. In an uncontrolled before-after design, participants showed significant improvement on a test of knowledge. However, only a few of the items on the knowledge test addressed the provision of help for activities of daily living, and simply improving knowledge about PCDC may not be sufficient to improve the quality of the care provided (Carparelli et al., 2024a). *iSupport* is an online training package for carers that is sponsored by the World Health Organization. It, too, includes a module about assisting with activities of daily living and the package is explicitly based on Kitwood’s account of PCDC (Pot et al., 2019). Much of what is taught in this module overlaps with issues highlighted in this study, such as maintaining the person’s independence, simplifying tasks, and keeping to routines. Studies have reported adaptations of the package to various cultural and geographic contexts, but there have been only two preliminary randomised controlled trials (Baruah et al., 2021; Teles et al., 2022). Only one outcome in each study showed significant benefit. The outcomes focused primarily on carer wellbeing and neither included a measure of the quality of care provided. There was also a relatively high rate of participant withdrawal in both studies, and also in the study of Gaugler et al. (2015). In summary, very few interventions have been developed to improve the quality of care provided by family members for activities of daily living, and there is no compelling evidence for their effectiveness in achieving that end.

This lack of evidence indicates the need for further work in developing interventions. The findings of the present study have several implications for this. As suggested in the Introduction, a useful starting point for developing an intervention is provided by the present study in the form of an account of actual carer practices that are categorised according to whether they are consistent or inconsistent with PCDC principles. The list of consistent practices shows how the principles of PCDC can be translated into specific activities that have been tried and tested in real life. These can be used as the basis for guidance for future family carers. The list of inconsistent practices is also important. As well as providing guidance about what to do, interventions need to cover what should be avoided.

Interventions also need to address the challenges of providing PCDC. As discussed earlier, the findings showed that the principles of PCDC can sometimes conflict with one another and with other considerations, such as safety and the carer’s own needs; and that carers sometimes need to weigh up these different considerations in a complex and changing situation. An intervention that provides simplistic rules and guidance derived from the principles of PCDC (e.g., you should always give the person a choice) may be unhelpful and lack credibility to family carers. Interventions should directly address these conflicts between different PCDC principles and other considerations, and include discussion about ways carers can resolve these conflicts, along with recognising that there may not always be a correct way of resolving the conflict.

### Limitations and future research

Information was not systematically collected about the degree of support participants provided to their partners. However, it was clear that many of the partners were still in the mild/moderate stages of dementia and relatively few were in the more advanced stages. It is likely that some of the issues that this study addressed change as the needs of the partner increase. Previous research has noted that the characteristics and relational dynamics of providing care and assistance change as cognitive impairment increases (e.g., La Fontaine et al., 2022; Samsi & Manthorpe, 2013). For example, one participant whose partner’s dementia was more advanced talked about the importance of learning to read the signs of what someone needed when they were no longer able to communicate verbally. Being able to read these signs should be considered part of the provision of good quality care.

The sample was almost exclusively White British. Previous research has highlighted the impact that cultural factors have on the way in which care is provided. For example, Chan et al. (2023) discuss how the emphasis on family harmony and the avoidance of conflict in Chinese culture may have influenced the participants in their study to prefer certain solutions to dilemmas arising in the provision of care, such as being more likely to allow their family member to act in ways that the participant considered incorrect or inappropriate (such as completing an activity of daily living incorrectly). In relation to the development of interventions to support family carers in assisting with activities of daily living, cultural factors should be addressed, and the intervention adapted accordingly when it is used in different cultural contexts.

The sample was self-selected from an online register of people interested in research. This may have resulted in findings in which care of poorer quality was underrepresented, because variables associated with volunteering for research may also be associated with providing better quality care. Carers who are under greater stress may be less likely to volunteer and find it more difficult to provide better quality care.

The data relied on self-report. Participants may have presented a more socially acceptable version of their actual caring behaviour; they may have lacked accuracy or insight; or they may not have been asked the right questions to cover all aspects of their performance. Previous research has suggested discrepancies between self-report and observational measures in this context. Small et al. (2003) found that, compared to observations, self-report underestimated the use of some communication adaptations, but overestimated others. Future research examining the quality of family care should also include observations of the care provided. In their observational study of activities of daily living assistance, Vikstrom et al. (2005) provided insights that were not described by the participants in the present study. They observed that some participants gave their partner a lot of time to think about what they were doing and to respond, whereas others would hurry them or take over if the response did not happen quickly enough. They also observed how some participants took care to prepare the environment, putting out relevant equipment and removing anything irrelevant, and discretely completing difficult steps of the task. On the negative side, instructions were sometimes too complex or unclear. They also observed that some participants did not provide sufficient supervision, leaving their partner to struggle with tasks and that this created an uncertain and unsupportive context for their partner.

More generally, our findings may have been limited by the broad scope of the questions asked in the interview. Focusing on particular aspects of assisting with activities of daily living or assisting with specific activities of daily living may provide further relevant insights. For example, Chan et al. (2023) focused on how family carers manage resistance and lack of co-operation and described various strategies used in relation to activities of daily living; specifically, promising the person something if they complied (sometimes without any intention to deliver on the promise); and a ‘foot-in-the-door’ approach (e.g., persuading them to use the toilet in preparation for getting them to shower).

### Conclusions

Family carers sometimes provide care that is inconsistent with the principles of PCDC and interventions to support them in improving the quality of care are required. Specific examples of good and poor quality care, provided by this and other studies, form a useful starting point for the development of practical guidance about good practices and practices to avoid. Interventions also need to acknowledge the challenges and complexities involved in providing care that is consistent with PCDC principles.

## Supplemental Material

Supplemental Material - Family carers and the provision of person-centred dementia care for activities of daily living Supplemental Material for Family carers and the provision of person-centred dementia care for activities of daily living by Chiara Carparelli, Jan R. Oyebode and Gerard A. Riley in Dementia
