# Supplementary material for: Family carers and the provision of person-centred dementia care for activities of daily living
Source: Dementia (London). 2025 Jan 6;25(1):3–24. doi: 10.1177/14713012241312266 (PMC12701084; doi:10.1177/14713012241312266)
Supplement: Supplemental Material - Family carers and the provision of person-centred dementia care for activities of daily living [file sj-pdf-1-dem-10.1177_14713012241312266.pdf]

Family carers and the provision of person-centred dementia care for activities of daily living: Supplementary material

Additional findings related to some beliefs and reasons that informed decisions that participants made about assisting with ADL, and to what guidance participants had received.

Reasons informing the provision of ADL assistance

For the purpose of developing interventions to support family carers to deliver good quality care, it is useful to explore the beliefs that inform the actions they take in relation to providing care. Interventions should address these beliefs, reinforcing valid beliefs relating to good quality care, and challenging those that lack validity and/or lead to care of poorer quality.

Reasons for promoting autonomy and independence

Numerous participants stated their belief that active engagement in ADL would help delay further loss of their partner's abilities:

Because it really is a case of if you don't use it, you lose it; and if he doesn't do, he is going to forget how to do it. (27)

Because with this disease, the minute you stop doing something, it’s lost in every area really. So, I encourage him to do as much as he can do on his own (22)

Another common reason was the promotion of the partner’s psychological well-being and self-esteem:

And equally I can understand how he could feel humiliated by needing that help because that's how I would feel, if it was me. So I'm constantly trying to help him maintain as much independence as he can for as long as he can. (6)

I think it’s important … for his own self-esteem that he can do some things, because sometimes he’ll say, ‘I can’t do anything’ and that’s simply not true. He can do loads and loads of things. (6)

I want him to do things which he knows- [so] he can say that ‘I did that’… for his own self esteem really. (10)

I think it's important for his well-being. (20)

One participant highlighted the importance of maintaining her partner’s independence as a way of sustaining the normality of their life together:

We've got to keep this normal life going for as long as we possibly can, I think…I think it’d be very easy to just give into this, and kind of slide into a whole different life, and neither of us wants that. (18)

Trying to maintain a sense of normality about their communication was important to some:

I try and make it a you and me thing. ‘Well, I've got [a few things to do] so I need to have a shower. So, who's going first? You want to go first, or do you want me to go for it? …. if you want to go now, I’ll do something else.’ So, it's a sort of try and make it as normal as possible. (21)

More specifically, fostering independence was perceived as a way of staying as partners in a normal relationship, and avoiding the relationship becoming one characterised by the giving and receiving of care:

> I've been trying to sort the finances for it and…I feel I need to keep him informed… We're together and that's part of being together - sort of doing things together and talking through things. (19)
>
> [I try] to make him just part of everything - which is how a relationship should be - that you're two people each deciding on things, trying to come together to do things together. (17)
>
> Well, again it's all part of being married this partnership and I don't want to get to the stage where she just completely backs out of living and to allow me to just control her whole life. (15)

Participants also saw maintaining independence as valuable in reducing the burden that fell on them:

> The more she can do independent with me not having to do things, the slightly easier it is for me. (1)
>
> The more she did, or the more she continued to do, the less I have to do it. (4)

For some participants providing choice was seen as a moral issue in line with the *valuing* principle of treating the care recipient with respect and in ways that everyone else expects to be treated:

> [Asked why she gave her partner a choice] To respect him. It's all about respect he still deserves respect. And I've had to drill into the carers 'don't treat…him like an old dear, a child'. You have to treat him with respect. (11)

Reasons for restricting autonomy and independence

Some participants believed that giving choice to their partner was pointless because of their mental state. One participant said that she did not give her husband a choice over what clothes to wear 'because he won't care' (12) and another did not give his wife any choice over what programmes to watch on TV:

> I suppose I'm a bit selfish now because it doesn't matter what she watches, it doesn't sink in, and she won't remember it anyway. (28)

Similarly, some participants believed that it served no purpose to inform their partner about errors they were making because of their cognitive difficulties:

> It's just not worth it…Absolutely no point because he won't remember next time so I'm just wasting my breath really. (12)
>
> Pointing out where something hasn't happened that she would like is just going to cause stress and not necessarily help her because some days she'll put it away correctly and some days she won't. (8)

Guidance about helping with ADL

Another aim of the study was to explore what advice and guidance participants had received about providing assistance for ADL. This information can help establish how much need there is for the development of interventions to help family carers deliver good quality care. A particular focus of interest was what participants knew about the concept of PCDC, and to what extent an understanding of this guided their delivery of care.

Participants had received or accessed information from a variery of sources, including printed and online information; other family members and friends; carer support groups (including online ones); charitable organizations; and health and social care services. Nearly all participants reported having received printed material about dementia, typically just after diagnosis. Although some found it helpful, many were critical of this material, feeling that it was given at the wrong time and did not address challenges that the couple were facing at the time it was received; that it was overwhelming in its quantity and emotional impact; and that any guidance it contained was too generalized, vague, and difficult to apply to their own situation. Much of the material appears to have been about the symptoms and course of dementia. Few participants could recall specific guidance about helping with ADL. Some mentioned learning about adapted equipment from printed material, and one participant said that he learned about the importance of routines, being organised, and better ways of communicating.

Family members, friends, and support groups were highly valued for the emotional, rather than the informational, support they provided. Again, only a few participants could recall advice about assisting with ADL. Some participants highlighted how useful they had found practical advice from charitable organizations, but this was about issues such as managing aggression rather than how to help with ADL.

Many participants were disappointed with the general lack of support that they received from health and social care services. They felt that, once the diagnosis had been given, they were generally abandoned by these services:

> You're on your own. There's no support, there's no ongoing care when you're diagnosed, you know. You can go to a health group and that's it, and literally you are cut adrift. (11)
>
> So, I mean we were given the diagnosis and we came out with a big bag full of leaflets. And we were virtually patted on the head and told to go way and get on with it. (25)

However, some examples of ADL advice that participants had received from health and social care staff were recalled, such as gaining the partner's attention before giving instructions, and being careful about correcting the partner because of his sensitivity to criticism.

In understanding this lack of guidance about ADL received by participants, it is important to note that there was a general reluctance from many participants to seek support and advice, which is likely to have contributed to the fact that, overall, the participants had received little guidance about ADL. Some did not feel that their partner had sufficiently advanced dementia to merit seeking advice. Support groups, for example, were viewed by some as intended for those dealing with more advanced dementia. One participant did not view herself as a 'carer' because her partner's needs were not sufficiently great:

> Although I obviously, you know talking to you, I can see that I probably do have quite a role in caring for him, I don't look at myself as a carer. (5)

Another factor contributing to unwillingness to seek help was a reluctance to burden family and friends with their problems:

> I don't want to burden them too much with the responsibility for, you know, having to care for me or for him. (6)

Reluctance to speak to family and friends was also motivated by a concern not to appear disloyal to their partner:

> It's all very hard and if you're speaking to someone who is not in that situation and doesn't realise the limitations that you have on your life and how it altered your life, it sounds a bit as if you're criticizing your partner and you're being disloyal; whereas if you're speaking to somebody who's been in that situation, they understand that you're not being disloyal, you're just trying to get it off your chest. (20)

For some, a major barrier was that seeking help felt like an admission of failure, or that they were being judged by others for asking for help:

> You don't want to be not coping, yeah, and asking for help because you failed, like you failed then really, so you do try and plod on really. (10)
>
> [Seeking support] is something I really need to do but you also feel slightly guilty that you're not coping. (14)

Some participants were sceptical about the usefulness of general advice, believing that it failed to address the complexity of the individual and their situation:

> Well, I think, when the original diagnosis came through you get a great wad of paper listing all the things that they suggest will work for the individual, but in my view, every person is very different you know all these words might work for a few people that probably wouldn't work for a whole lot more. (22)
>
> How hopeless, it is to read stuff. Everybody is different, the patient is different, the circumstances are different, the care is different (1)

As part of the exploration of what guidance they had received, participants were also asked about their knowledge of the concept of 'person-centred care'. Only 13 of the 30 participants stated that they had heard the term before. Only 6 of these 13 had discussed or read about its relevance to dementia care, with others knowing about the concept from another context such as a different branch of healthcare. Only one participant (11) recalled a discussion with

health and social care staff about the principles of PCDC, and this was in the context of making a decision about which residential home her partner should move into. Participants were also asked whether they intentionally tried to apply what they had learnt about PCDC to their care practice. Only two participants said that they did this.

The participants who had heard about PCDC were also asked about their understanding of the term. All but two (11 and 16) gave an explanation in terms of just one of the VIPS principles Most focused on the *individual* principle and the importance of finding out what the person needed or wanted, and tailoring the care to make sure this need/want was met.

> Everybody with dementia…[is] completely different - so you've got to tailor [the care] for that person's needs. (26)
>
> It means every person who needs care has different needs and that you have to know what those needs are and try and meet them. (5)

Some participants gave an explanation in terms of the *valuing* principle:

> I assume it's to do with my husband, letting him make decisions as best he will and encouraging him to do so. (25)
>
> Regardless of whether it's dementia, it could be a stroke, or whatever you're you're still in a human being, who should be respected and helped to get whatever quality of life, you can. (11)

Participant 11 and 16 had more detailed knowledge of the concept and gave an explanation that covered both the *individual* and *valuing* domains.

Two participants described PCDC as being about subjugating their own needs to those of their partner. One participant explained it in terms of "making sure her needs come first".

He described as “selfish” his act of taking her to an event that he wanted to go to and that she would not have gone to, had there been someone to look after her.
